# Practical guide to building machine learning-based clinical prediction models using imbalanced datasets

**DOI:** 10.1136/tsaco-2023-001222

**Published:** 2024-06-12

**Authors:** Jacklyn Luu, Evgenia Borisenko, Valerie Przekop, Advait Patil, Joseph D Forrester, Jeff Choi

**Affiliations:** 1 Stanford University, Stanford, California, USA; 2 Department of Surgery, Stanford University, Stanford, California, USA

**Keywords:** guideline, tracheostomy, epidemiology, Models, Statistical

## Abstract

Clinical prediction models often aim to predict rare, high-risk events, but building such models requires robust understanding of imbalance datasets and their unique study design considerations. This practical guide highlights foundational prediction model principles for surgeon-data scientists and readers who encounter clinical prediction models, from feature engineering and algorithm selection strategies to model evaluation and design techniques specific to imbalanced datasets. We walk through a clinical example using readable code to highlight important considerations and common pitfalls in developing machine learning-based prediction models. We hope this practical guide facilitates developing and critically appraising robust clinical prediction models for the surgical community.

## Introduction

Despite surging interest in machine learning-based clinical prediction models, few are implemented at the bedside.^1^ The implementation gap may be attributable to studies that do not follow standard reporting guidelines, provide insufficient performance evaluation (eg, reporting only area under the receiver operating characteristic curve (AUROC) for model discrimination, not reporting calibration), and lack implementation practicality (eg, not building usable models).^2–4^

Many clinical prediction models involve predicting rare, high-risk events: these constitute imbalanced datasets.^5 6^ Designing and interpreting models using imbalanced datasets are fraught with pitfalls: expert involvement is essential. We aimed to highlight key study design and analytic considerations for building clinical prediction models using imbalanced datasets. Using an example clinical problem, this practical guide shares common pitfalls and readable code to elevate trauma surgeons’ ability to build useful models and appraise prediction model literature.

### Foundational machine learning and prediction model principles

#### Dataset splitting

A useful clinical prediction model must provide reliable predictions and be generalizable. Overfitting is a common phenomenon where a model outputs reliable predictions for the population it was built on, but is not generalizable for other populations (ie, high variance).^7^

A dataset is commonly split into three mutually exclusive training, validation, and test sets. The training set derives initial model parameters (eg, beta coefficients in logistic regression) that specify how inputs would be mapped to outputs. The validation set is used to fine-tune the model (many machine learning models have hyperparameters, such as lambda coefficient in logistic regression) to optimize performance and generalizability. The fine-tuned model’s performance is evaluated on the held-out test set. The gap between training/validation and test set performance can highlight a prediction model’s generalizability.

Other dataset splitting strategies (eg, training/test set split, cross-validation) are possible. Dataset dimensions (the number of subjects vs. the number of predictors per subject), algorithm choice, and other task-specific considerations should guide dataset splitting strategies, yet the importance of estimating model generalizability is universal.

### Algorithm choice: flexibility, interpretability, and the black box problem

Choosing the appropriate machine learning algorithm for a prediction model must consider the model flexibility–interpretability tradeoff. Model flexibility describes the complexity of relationships that can be mapped between predictors and the output. Flexible models can map complex, non-linear relationships but risk overfitting and may not be generalizable (ie, high variance, low bias). Model interpretability characterizes whether humans can understand how a model derives predictions.^8 9^ In general, flexible models are less interpretable: we highlight the flexibility–interpretability tradeoff using three common machine learning models.

### Multivariable logistic regression

Multivariable logistic regression has high interpretability: the beta coefficients associated with each predictor can be converted into ORs. However, logistic regression has limited flexibility because it models a linear relationship between each predictor variable and the logit of the outcome ( $logit\left(p\right)=log\left(\frac{p}{1-p}\right)$ , where p=probability of the outcome). Regularization (ridge, LASSO (least absolute shrinkage and selection operator), elastic net) can mitigate overfitting by adding a penalty to hyperinflated coefficients.

### Decision tree

**Figure 1:**
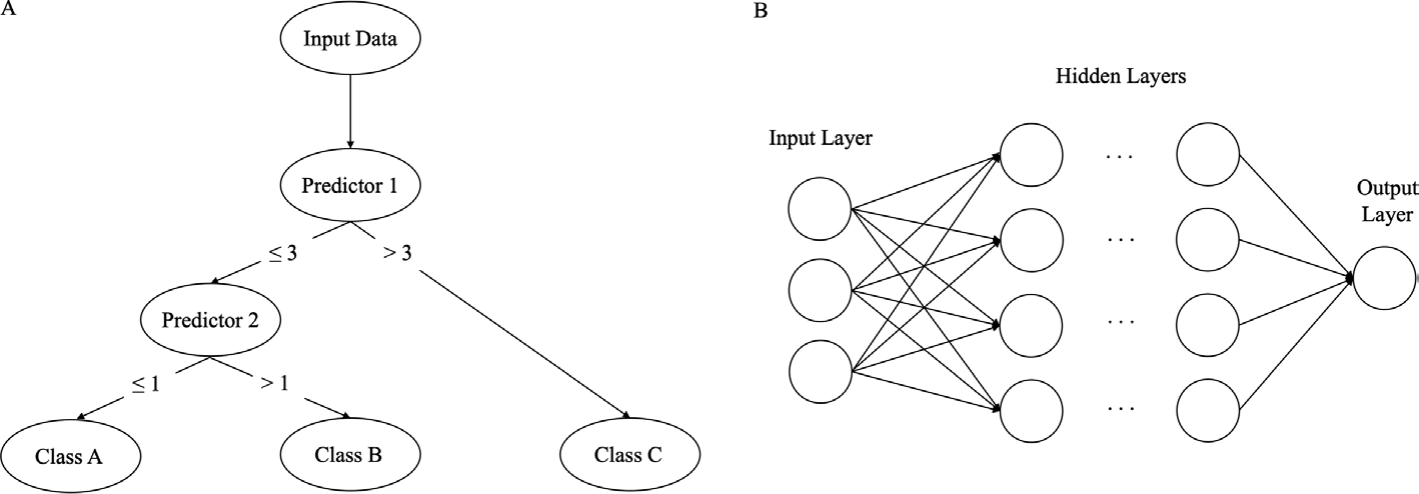
(A)Simple decision tree architecture. At the top of the tree (‘root node‘), the input data are split into smaller partitions (nodes) based on various decision points, until the final classification (‘leaf node‘) is reached. (B Architecture of a simple multilayer perceptron. The first ‘input layer’ comprises predictors. Multiple hidden layers calculate a weighted sum of the previous layer’s neurons, transform the calculation using a non-linear activation function, and pass the result onto subsequent layers in the network. The final output layer produces a prediction.

Tree-based models output predictions using decision rules based on predictor-specific thresholds (figure 1A). The tree branch-like model structure facilitates interpretability using visual diagrams and is more flexible compared with logistic regression (non-linear predictor–outcome relationships can be mapped). However, if overfit, small changes in study population characteristics can drastically change tree structures. Bagging and random forest can address overfitting by aggregating the outputs of multiple individual decision trees.

### Deep neural networks

Deep neural networks use multiple layers of neurons (units of simple computations and non-linear transformations) to make predictions (figure 1B). Deep neural networks are highly flexible models that can map complex relationships between predictors and an outcome. However, understanding how a prediction was made is challenging. Poor interpretability remains a challenge in adopting deep neural network-based clinical prediction tools. ‘Black box’ models lack quality assurance, as it is difficult to identify and correct errors in models’ performance and biases.^10^ Lack in transparency and accountability undermines trust from clinicians who use and patients who are impacted by models’ performance. Justifying medical decisions in intelligible ways is an ethical requirement of practitioners, which may be hindered by ‘black box’ models. As a result, interpretability in ‘black box’ models remains a significant implementation barrier.^11 12^

### Model evaluation

Most published prediction models only report discrimination metrics. Although AUROC is the most commonly reported discrimination metric, AUROC is often misleading and uninformative for imbalanced datasets.^13^ Both discrimination and calibration metrics must be reported.

### Discrimination

Discrimination describes a model’s ability to separate outcomes into classes (eg, if binary outcome is mortality: mortality vs. survival). Most clinical prediction tasks involve imbalanced datasets with unevenly distributed outcome classes (eg, mortality prediction: many patients do not experience mortality).^14^ Depending on the prediction task, different discrimination metrics are important to optimize. Table 1 highlights common discrimination metrics and important considerations for imbalanced datasets.

**Table 1:** Common discrimination performance metrics

Metric	Description
Accuracy	Proportion of correct predictions, but can be misleading for imbalanced datasets because a model will be biased toward the majority class. In a population with 1% mortality, a model that always predicts a patient will experience mortality would have 99% accuracy.
Precision (positive predictive value)	Proportion of true positives among those predicted to be positives. Maximizing precision is useful when minimizing false positives is important (eg, if an invasive intervention would be performed for patients predicted to be positive).
Recall (sensitivity)	Proportion of positive samples that are correctly classified (**‘**positive in disease’). Maximizing recall is useful when screening for critical diagnoses.
Specificity	Negative class version of recall; proportion of negative samples that are correctly classified (‘negative in health’).
F1 score	Weighted average of precision and recall. Weighted F1 scores can assign different weights to precision and recall, and are helpful for imbalanced datasets where the cost of predicting false negatives and false positives is unequal.
Area under the receiver operating characteristic curve (AUROC)	Proportion of a randomly chosen positive that would be ranked before a randomly chosen negative. This definition is not intuitive for clinical decision-making and is often misinterpreted. An AUROC of 0.5 is a random classifier, but even with high AUROC values, AUROC is often misleading and falsely optimistic metric for imbalanced datasets because it equally weighs false positives and false negatives.
Area under the precision–recall curve (AUPRC)	Preferable to the AUROC when dealing with imbalanced datasets. The ‘baseline’ AUPRC notes the proportion of positives: the baseline would be 0.5 for a perfectly balanced dataset but 0.1 for an imbalanced dataset where the ratio of positives to negatives is 1:10.
Average precision	Weighted mean of precision scores for all decision thresholds (most models output the probability of a sample having an outcome, and the thresholds delineating positive vs. negative outcome can be varied).

### Calibration

**Figure 2:**
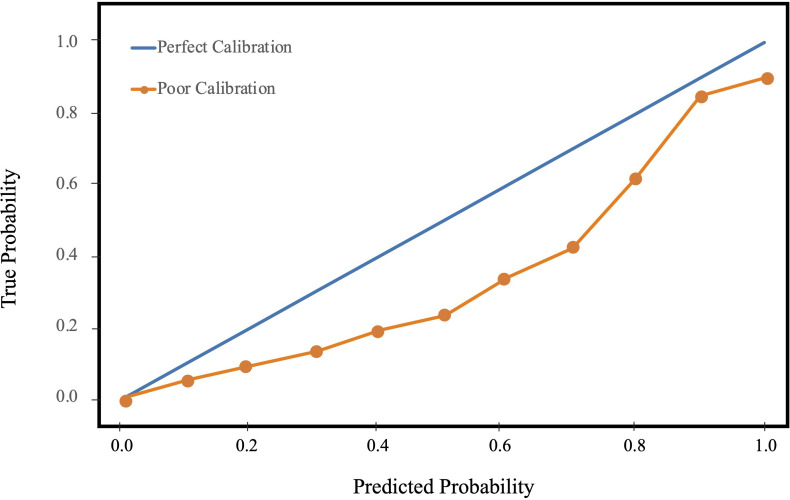
Example of a calibration plot.

Despite the importance of calibration for evaluating the performance of prediction tools, few studies assess model calibration.^15^ Calibration measures the closeness of a model’s predicted probability to the underlying probability distribution of the study population. Models with high discrimination performance can systematically overpredict or underpredict risk and hinder real-world adoption. Calibration can be visualized with calibration curves which plot predicted probability of an event against the true probability within the study population.^16^ A perfectly calibrated model would yield a 45° line in the calibration curve (figure 2).

### Clinical relevance

Even with high discrimination and calibration performance, a prediction model may not be necessarily useful unless it can guide actionable decisions based on the prediction. For example, a high-performing mortality prediction model would not be useful if knowing a patient has high risk of mortality does not guide decision-making. Decision curve analysis is one way to gauge clinical relevance. Decision curve analysis helps visualize a clinical ‘net benefit’ for making a decision based on a prediction model (eg, performing an operative intervention) compared with default strategies of treating all or no patients, taking into account clinicians’ preferences (ie, being more conservative vs. more aggressive in deciding to intervene).^17 18^

### Methods to address dataset imbalance

Two general categories to address dataset imbalance comprise data-level methods and algorithm-level methods.^19^ Data-level methods aim to artificially balance the skewed class distribution by resampling the original dataset. This can be done by oversampling the minority class, undersampling the majority class, or a combination of both.^20^ Algorithm-level methods use cost-sensitive learning, where an algorithm assigns more weight to a misclassification (eg, assigning a false negative prediction a higher weight compared with a false positive prediction).

### Example prediction task and dataset

We provide a clinical example to highlight key considerations when developing and validating a prediction model using an imbalanced dataset. This example is not meant to be a standalone observational study—we do not cover all components suggested by the Transparent Reporting of a multivariable prediction model for Individual Prognosis Or Diagnosis guideline.^15^ Rather than the nuances of the clinical question, actual model performance, or clinical utility, we hope to highlight *key study design decision points* using a familiar database and share common prediction model programming code using an iPython notebook file (online supplemental file). Table 2 explains common technical terms.

10.1136/tsaco-2023-001222.supp1 Supplementary data

**Table 2:** Explanation of technical terminology

Terminology	Explanation
Hyperparameter	‘Adjustable’ variables that dictate how model parameters are estimated. These configurations are specified by the practitioner prior to model learning and cannot be learned from the data.
Model fine-tuning	Fine-tuning describes the iterative process of training prediction models using different sets of hyperparameter values. Fine-tuning using the validation set facilitates identifying hyperparameter configurations that yield the highest performance, which can be evaluated on a held-out test set.
Regularization	Beta coefficients are estimated by minimizing loss functions. Regularization techniques (ridge, LASSO, elastic net) can mitigate overfitting models to specific datasets and improve generalizability by penalizing high-value coefficients.
Bagging and random forest	Bagging is a general model-averaging approach used to reduce variance and prevent overfitting models to noisy data. A random forest algorithm applies bagging to decision trees by combining the outputs of multiple decision trees trained independently on random samples of the training data.
Feature engineering: one-hot encoding and min–max normalization	One-hot encoding (converting categorical variables into unique binary numbers) is used as most models require numerical input and output. Min–max normalization (rescaling continuous variables to fit within the range (0–1)) can improve model performance by eliminating impact of different scales.
Gradient-boosted decision tree: maximum tree depth, learning rate, subsample fraction, regularization strength, and early stopping	A decision tree is a flow chart-like structure, where the input data are split into smaller partitions (nodes) based on various decision points, until the final classification (‘leaf node’) is reached. The maximum tree depth limits the number of splits that can be performed in a decision tree. Learning rate determines the speed at which a model will update its parameters. Subsample fraction is the portion of training data used to create an individual tree. Regularization strength controls the size of penalties given to large beta coefficients. Early stopping is a technique used to mitigate overfitting whereby the learning process is halted if the model performance does not improve.
Multilayer perceptron: dense–dropout–batch normalization layers, gradient descent, Adam optimization, epochs and batch size	Dense (each neuron in the layer receives input from every neuron in the previous layer), dropout (randomly excludes some inputs to prevent overfitting), and batch normalization (normalizes and rescales input units to accelerate training) layers are common types of layers found in a multilayer perceptron. Gradient descent with Adam optimization (method used to learn model parameters while minimizing the cost function), epochs (the number of complete passes through the training data), and batch size (number of samples evaluated in a single pass before updating parameters) are additional model learning hyperparameters.
SHapley Additive exPlanations	Technique used to elucidate the marginal contribution of each predictor for deriving a model’s output.

LASSO, least absolute shrinkage and selection operator.

**Table 3:** Discrimination performance of candidate prediction models

Performance metric	LASSO regression	XGBoost	MLP
Recall	0.900	0.900	0.900
Precision	0.042	0.096	0.053
F1 score	0.158	0.173	0.100
Accuracy	0.836	0.853	0.724
AUROC	0.937	0.943	0.908
Average precision	0.254	0.261	0.231

In machine learning literature, the standard is to report performance metrics to the thousandth decimal place.

AUROC, area under the receiver operating characteristic curve; LASSO, least absolute shrinkage and selection operator; MLP, multilayer perceptron; XGBoost, gradient-boosted decision tree.

*The prediction task:* identify patients who undergo tracheostomy after traumatic injury.

*Dataset:* We queried the 2017 National Trauma Data Bank for adults (age ≥18 years) hospitalized with traumatic injury. We excluded patients who underwent emergent tracheostomy performed within 24 hours of hospitalization. Emergent tracheostomy may be performed in response to specific injury patterns (eg, multiple facial bone fractures); this constitutes a population distinct from patients who undergo tracheostomy in anticipation of prolonged ventilatory support and may benefit from early detection to mitigate harms of prolonged intubation.

*Outcome:* probability of undergoing tracheostomy.

*Toolkits and packages:* Our analysis used two popular Python toolkits, Scikit-learn (beginner-friendly, wide range of machine learning algorithms used for predictive modeling) and TensorFlow (open-source framework used for large-scale deep learning techniques). Of note, Pytorch is an alternative to TensorFlow, but TensorFlow offers better visualizations for tracking model training.

### Feature engineering

Candidate model predictors comprised demographic, hospital, and hospitalization characteristics that could reasonably be associated with undergoing tracheostomy. We one-hot encoded categorical predictors and min–max normalized continuous predictors. Standardizing or normalizing continuous predictors is important for many models. We excluded predictors with >10% missingness and predictors capturing events after undergoing tracheostomy (eg, hospital length of stay).

The National Trauma Data Bank lists up to 40 International Classification of Diseases, 10th revision (ICD-10) diagnosis codes per patient, which detail injuries and baseline health conditions. The ICD-10 diagnosis codes comprise a seven-character ontology where successive characters specify more detailed diagnoses. We reshaped all ICD-10 diagnosis codes seen within our study population into binary predictors. To reduce dimensionality, we condensed all ICD-10 diagnoses into the first three characters and removed ICD-10 codes with <1% prevalence in the study population. Without dimensionality reduction, the dataset would have been sparse (eg, many predictors with ‘zero’ values) and risked overfitting and increased computational complexity. To standardize inputs and compare performance of different algorithms, we excluded patients with any missing data among the final predictors (multilayer perceptrons cannot use missing values for training).

Among 281 529 patients in the study cohort, 4815 (1.7%) underwent tracheostomy. From 1072 candidate predictors, after excluding clinically irrelevant predictors (N=35), those with >10% missingness (N=11) and rare ICD-10 diagnoses (N=892), 344 predictors constituted the final list of model predictors.

We divided our study population 80:20 into training:testing cohorts, stratified by the number of patients who underwent tracheostomy to ensure both cohorts comprise a representative proportion of cases.

### Model development

#### Overview

We trained three prediction models using LASSO regression, gradient-boosted decision trees (XGBoost), and multilayer perceptron. Each model used fivefold cross-validation and performed grid or random search to tune hyperparameters. Models were trained to maximize average precision (when training, the developer must choose a performance metric to optimize). To account for class imbalance, model loss functions accounted for class weights (inverse of outcome class probabilities).

#### LASSO regression

LASSO is a regularization technique (other options include ridge, elastic net) that removes less significant predictors (ie, small beta coefficients) from the model. The final LASSO model had a lambda (a hyperparameter that denotes regularization strength) of 0.1, and comprised 123 of the 344 original predictors.

#### Gradient-boosted decision tree

XGBoost is a decision tree algorithm that uses gradient boosting, which places numerous decision trees in sequence to improve performance. Our final XGBoost model used 300 boosting rounds with the following hyperparameters: maximum tree depth of 3, learning rate of 0.1, subsample fraction of 0.8, and regularization strength of 0. We used early stopping with 10 rounds.

#### Multilayer perceptron

**Figure 3:**
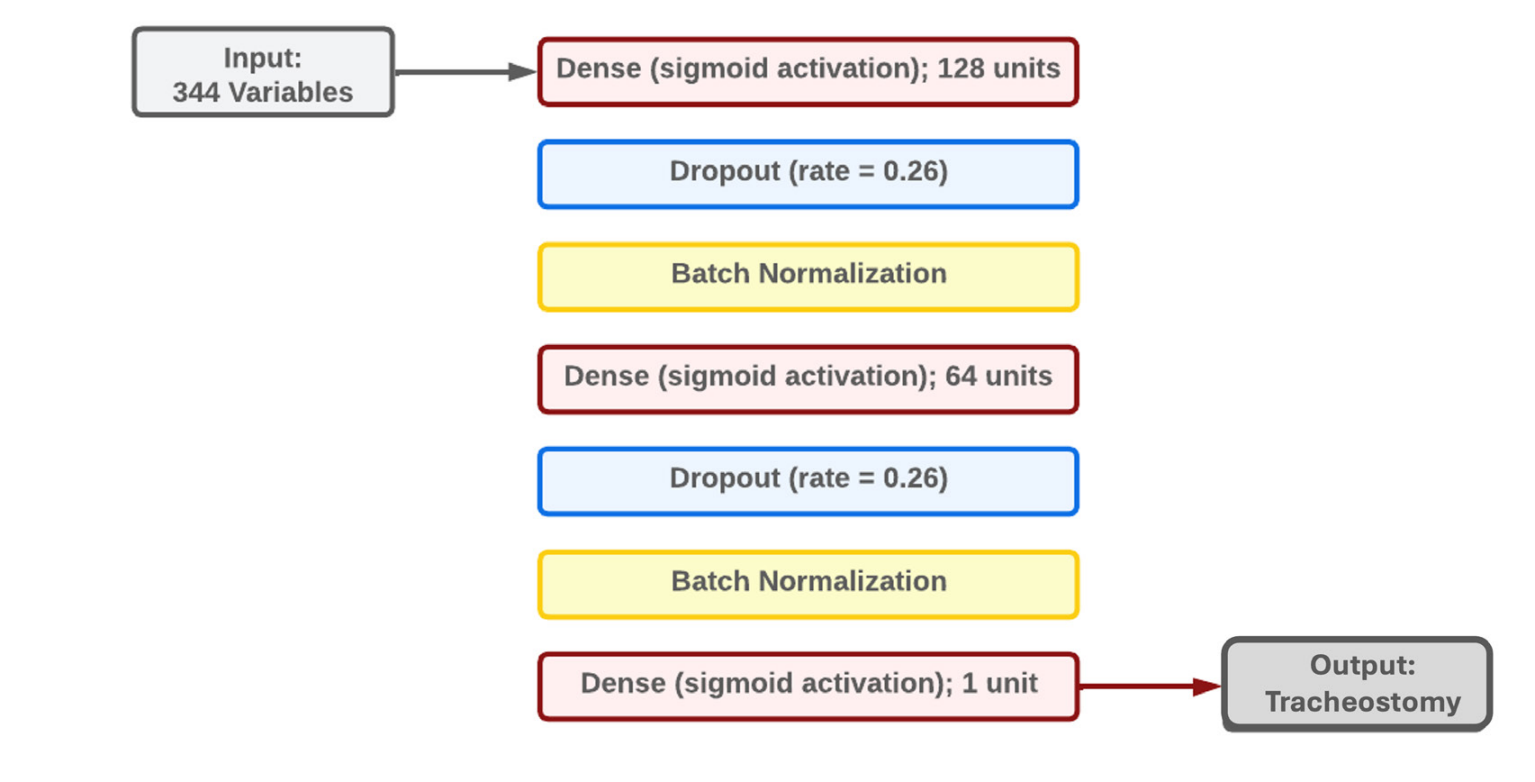
Multilayer perceptron model architecture.

Figure 3 details the final multilayer perceptron model architecture. The model comprised two consecutive series of dense–dropout–batch normalization layers, with a final fully connected layer. We performed gradient descent using Adam optimization and trained the model for 30 epochs with a batch size of 100.

#### Selecting best model using discrimination metrics

We evaluated the three models’ discrimination performances on the test set (table 3). We defined the best model as the one with the highest specificity with a minimum 0.90 recall, to minimize both false negatives (important quality for prediction tools that ‘screens high-risk patients’) and false positives (ie, to mitigate suggesting ‘unnecessary’ tracheostomies). The XGBoost model outperformed other models across most metrics and was the highest-performing model with a 0.852 specificity at a 0.900 recall threshold.

### Model evaluation

#### Calibration

**Figure 4:**
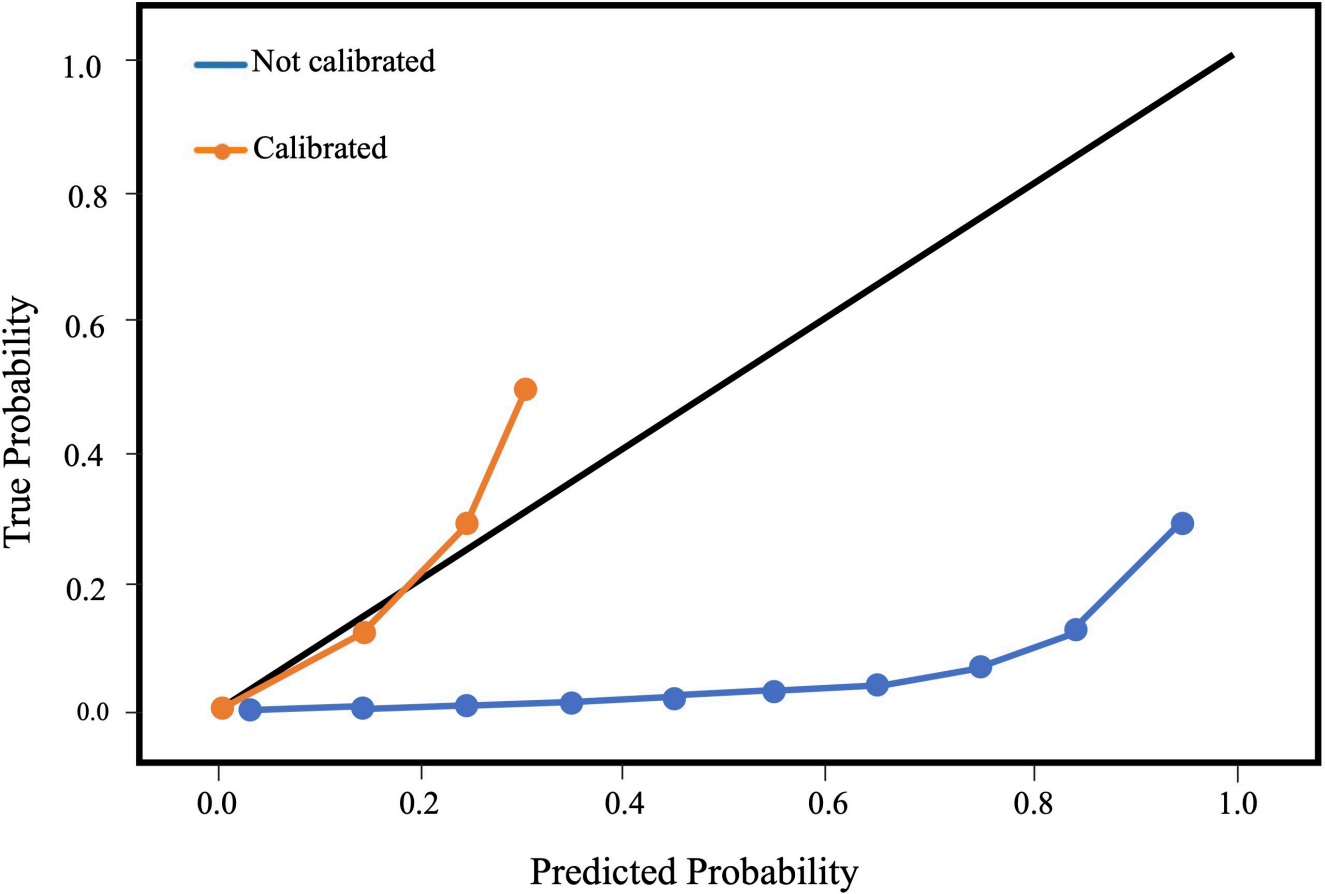
Gradient-boosted decision tree model calibration plot. The black diagonal line indicates a perfectly calibrated model, where the predicted probability of tracheostomy mirrors the observed probability of tracheostomy across the study population. The calibrated model’s line ends at approximately 0.31, because 0.31 was the highest predicted probability of undergoing tracheostomy after calibration.

The uncalibrated XGBoost model displayed a negative calibration slope distant from the diagonal (figure 4). After calibration, the calibration slope was closer to the diagonal, indicating closer approximation between predicted and true probabilities of undergoing tracheostomy. Our original XGBoost model had high discrimination performance (AUROC 0.94, recall 0.90) yet systematically overestimated the probability that a patient would undergo tracheostomy. Deploying the original XGBoost model without recalibrating would have resulted in the model suggesting tracheostomy ‘too aggressively’. Calibration analysis allowed us to recalibrate and produce a model whose predictions better align observations from a nationally representative, real-world dataset.

### Predictor importance

**Figure 5:**
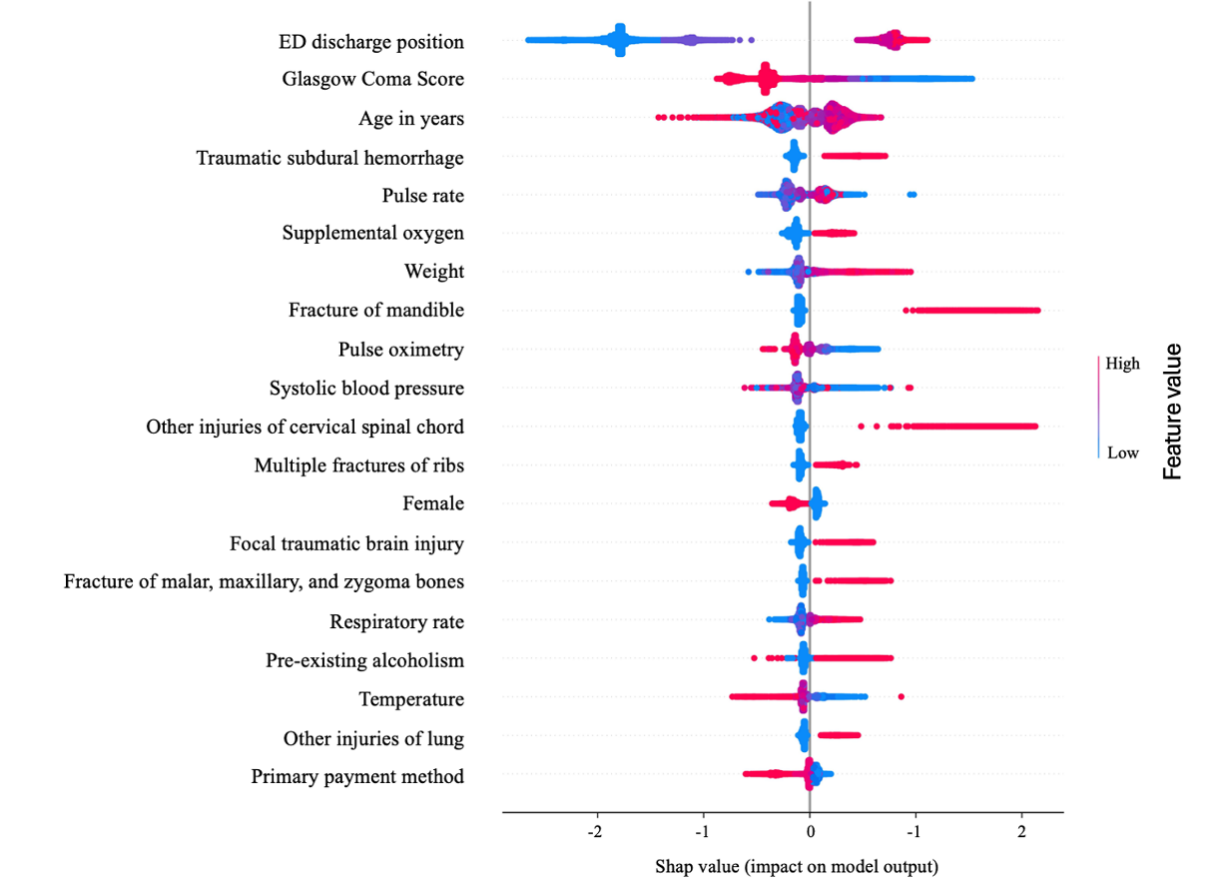
Tracheostomy predictors for XGBoost. SHAP summary plot of top 20 predictors for each model. Each point represents a value for each predictor of tracheostomy, whereas color indicates the predictor’s value. ED, emergency department; SHAP, SHapley Additive exPlanations; XGBoost, gradient-boosted decision tree.

We conducted SHAP (SHapley Additive exPlanations) analysis to elucidate the marginal contribution of each predictor for deriving the output.^21^ SHAP analysis confirmed that the predictors contributing most to the model’s predictions were sensible (figure 5).

### Data resampling

To evaluate whether reducing class imbalance would improve discrimination performance, we retrained our XGBoost model on a resampled dataset created using Synthetic Minority Class Oversampling Technique-Edited Nearest Neighbors (SMOTE-ENN). SMOTE oversamples the minority class, whereas ENN undersamples the majority class within the dataset. Using the same set of hyperparameters on resampled data, the discrimination performance was lower (recall: 0.900; precision: 0.063; accuracy: 0.769; F1 score: 0.118; AUROC: 0.914; average precision: 0.200).

## Conclusion

Designing useful clinical prediction tools is challenging; using imbalance datasets requires developers and readers to understand important study design and analytic nuances. We hope our overview and case example with publicly available code highlight important principles and can help our community develop meaningful clinical prediction tools.
