# Supplementary material for: Practical guide to building machine learning-based clinical prediction models using imbalanced datasets
Source: Trauma Surg Acute Care Open. 2024 Jun 12;9(1):e001222. doi: 10.1136/tsaco-2023-001222 (PMC11177772; doi:10.1136/tsaco-2023-001222)
Supplement: Supplementary data [file tsaco-2023-001222supp001.pdf]

A Guideline to Facilitate Building Machine Learning-Based Clinical Prediction Models Using Imbalanced Datasets

This notebook serves as a replicable guide to create machine learning based prediction tools for rare and high-risk clinical events, known as imbalanced datasets.

Data Pre-processing

First we import libraries and packages.

```
## import necessary libraries
import numpy as np    # mathematical operations using arrays
import pandas as pd   # data wrangling and manipulation
import os             # interactions with operating system
import random         # create pseudo-random number generators
import time           # time-related functions
import matplotlib.pyplot as plt   # create visualizations
from collections import Counter   # keep track of elements and their count


## import machine learning related libraries and packages
from sklearn.model_selection import train_test_split, StratifiedKFold, GridSearchCV
from sklearn.metrics import classification_report, confusion_matrix, f1_score
from sklearn.metrics import RocCurveDisplay, PrecisionRecallDisplay, ConfusionMatrixDisplay
from sklearn.metrics import roc_curve, precision_recall_curve, auc, average_precision_score, accuracy_score

import tensorflow as tf
```

Next we import the data.

A depiction of the directory and file structure is shown below. We created a group titled `trach_group` within our Shared Google Drive. The datafile is located in a subfolder called `data`, and the file is titled `nn_input__missing_lowprev_removed.csv`.

```
```
Shared Google Drive
│
└───trach_group
│   │   prediction_tool_code.ipynb
│   │
│   └───data
│   │   nn_input__missing_lowprev_removed.csv
│   │   ...

```
```

In order to import the datafile into this iPython notebook, users must do the following:

1. Create a group within the shared Google Drive
2. Create a subfolder called `data` within the group
3. Upload the datafile into the subfolder `data`

```
# connect google drive
from google.colab import drive  # allows for interaction with google drive
drive.mount('/content/drive')

# denote path to shared Google Drive
ROOT = '/content/drive/Shareddrives/trach_group'

# upload the datafile into dataframe
df = pd.read_csv(os.path.join(ROOT, 'data/nn_input__missing_lowprev_removed.csv'))

# drop unnecessary variables
df = df.drop(["inc_key", "Minutes_to_first_TRACHEOSTOMY","hispanic"],axis=1) # hispanic has a lot of missing values
df.index.size
```

Next, we perform feature engineering, where we pre-process the data in order to create suitable input for our machine learning models.

```
## shaping predictors
from sklearn.preprocessing import MinMaxScaler # scale continuous variables to fit within range [0,1]

# min-max transform certain continuous variables
scaler = MinMaxScaler()
df[["ageyears","sbp","pulserate","temperature","respiratoryrate","pulseoximetry","height",
    "weight","gcseye","gcsverbal","gcsmotor","totalgcs"]] = scaler.fit_transform(df[["ageyears","sbp","pulserate","te
    "weight","gcseye","gcsverbal","gcsmotor","totalgcs"]])

# one hot encode non-ordinal categorical variables
pd.get_dummies(data = df, columns = ['primarymethodpayment', 'eddischargedisposition', 'teachingstatus',"hospitaltype

# check for null values
pd.set_option('max_rows', 99999)
df.isna().sum()

# remove null values
df_no_missing = df.dropna()
df_no_missing.shape
```

Finally, we create `X` (our feature matrix) and `Y` (the variable we are trying to predict, which is elective tracheostomy. We split `X` and `Y` into training and testing sets.

```
# create train-test split (80/20) and stratify by y (elective_trach)
y = df_no_missing['elective_trach']
X = df_no_missing.drop(columns=['elective_trach'])
x_train, x_test, y_train, y_test = train_test_split(X, y, test_size=0.2, stratify=y, random_state=5)

print(f"X_train shape {x_train.shape}")
print(f"y_train shape {y_train.shape}")
print(f"X_test shape {x_test.shape}")
print(f"y_test shape {y_test.shape}")
```

Since we are dealing with an imbalanced dataset, we want to know the proportion of patients who do and do not undergo elective tracheostomy, called class weight. We save this information to adjust our models later.

```
# define positive class scaling (class weight)
count = Counter(y_train)
scale_pos_weight = count[0] / count[1]
print(scale_pos_weight)

class_weights = {0:1 , 1:scale_pos_weight}
```

Model Development

Here we train, tune, and evaluate 3 machine learning models: logistic regression, multi-layer perceptron, and XGBoost. For each model, we used 5 fold cross validation to determine which hyperparameters yielded the highest performing model. We then evaluated each model's discrimination performance using various metrics.

Logistic Regression with Lasso Regularization

```
from sklearn.linear_model import LogisticRegression

# define dictionary to tune C, the regularization strength
param_grid = {'C': [0.01, 0.1, 1, 10, 100, 1000]}

# perform 5-fold cross validation to tune hyperparameters
log = LogisticRegression(penalty='l1', solver='liblinear', class_weight = class_weights)
cv = StratifiedKFold(5, shuffle=True, random_state=5)
model = GridSearchCV(log, param_grid, cv = cv, refit = 4, verbose=3, n_jobs = -1, scoring = 'average_precision')
%time model.fit(x_train, y_train)
```

```
# print best hyperparameters
best_params = model.best_params_
best_params
```

```
{'C': 0.1}
```

```
# compute class probabilities
y_pred = model.predict_proba(x_test)
```

Discrimination Performance

```
## evaluate discrimination performance

# report discrimination metrics at different probability cutoffs
# (positive label assigned if positive class probability > cutoff)

cutoffs = np.arange(0.1, 1, 0.1)
target_names = ['negative', 'positive']

for cutoff in cutoffs:
  print('Cutoff: ', round(cutoff, 2))
  print(classification_report(y_test, np.where(y_pred[:,1] > cutoff, 1, 0), target_names=target_names))
```

```
# get roc
y_pos = y_pred[:, 1]
fpr, tpr, thresholds = roc_curve(y_test, y_pos)

# get index of sensitivity closest to 90+
indices = np.where(tpr >= 0.9)[0]
i = indices[tpr[indices].argmin()]

# calculate metrics
auroc = auc(fpr, tpr)
sens = tpr[i]*100
spec = (1 - fpr[i])*100
cutoff = thresholds[i]
y_pred_bin = (y_pred[:,1] >= cutoff).astype(int)
acc = accuracy_score(y_test, y_pred_bin)*100
avg_prec = average_precision_score(y_test, y_pos)*100
f1 = f1_score(y_test, y_pred_bin)*100

print("Recall of at least 90:  ", round(sens, 3))
print("Specificity:\t\t", round(spec,3))
print("AUROC:\t\t\t", round(auroc, 3))
print("Probabitiy cutoff:\t", round(cutoff, 3))
print('Accuracy:\t\t', round(acc, 3))
print('Average Precision:\t', round(avg_prec, 3))
print('F1-score:\t\t', round(f1, 3))



# plot ROC curve
plt.plot(fpr, tpr, label='Linear Classifier (AUROC = %0.2f)' % (auroc))
plt.xlim([-0.05, 1.05])
plt.ylim([-0.05, 1.05])
plt.axhline(y=sens/100, color='r', linestyle='--', linewidth=1)
plt.axvline(x=1-spec/100, color='r', linestyle='--', linewidth=1)
plt.xlabel('False positive rate')
plt.ylabel('True positive rate (Recall)')
plt.legend(loc="lower right")
plt.show()


# plot Precision-Recall curve
precision, recall, _ = precision_recall_curve(y_test, y_pos)
indeces = np.where(recall >= 0.9)[0]
i = indeces[recall[indeces].argmin()]
rec_at_cutoff = recall[i]*100
prec_at_cutoff = precision[i]*100

plt.plot(recall, precision, label='linear classifier (AP = %0.2f)' % (avg_prec))
plt.xlim([-0.05, 1.05])
plt.ylim([-0.05, 1.05])
plt.axhline(y=prec_at_cutoff/100, color='r', linestyle='--', linewidth=1)
plt.axvline(x=rec_at_cutoff/100, color='r', linestyle='--', linewidth=1)
plt.xlabel('Recall')
plt.ylabel('Precision')
plt.legend(loc="lower right")
plt.show()
```

Multi-layer Perceptron (MLP)

```
from scikeras.wrappers import KerasClassifier       # allows interaction with tensorflow and sci-kit learn
from sklearn.model_selection import cross_val_score # evaluate a score by cross-validation
from keras.models import Sequential                 # create a sequence of layers
from keras.layers import Dense, Dropout, BatchNormalization # types of layers in MLP
from keras.callbacks import EarlyStopping           # stops model training early if performance hasn't improved
```

```
## Run k-fold cross validation to tune hyperparameters related to MLP architecture

def run_cv(n_iters, n_folds):
  """
  Custom function to tune MLP's hyperparameters specific to the model's architecture.
  Scores each fold based on average precision, to account for class imbalance.
  ====
  INPUTS
  - n_iters (integer): number of combinations to test
  - n_folds (integer): number of folds to run cross-validation
  OUTPUT
  - res: list of average precision scores
  """
  res = []
  for i in range(n_iters):
    # select random combination of optimizer, activation fn, and dropout rate
    optimizer = np.random.choice(['Adam', 'Adagrad'])
    activation = np.random.choice(['relu', 'tanh', 'sigmoid'] )
    dropout_rate = np.random.choice([0.2, 0.3, 0.4, 0.5])

    # create MLP model
    def create_mlp():
        model = Sequential()
        model.add(Dense(128, input_dim=x_train.shape[1], activation=activation))
        model.add(Dropout(dropout_rate))
        model.add(BatchNormalization())
        model.add(Dense(64, activation=activation))
        model.add(Dropout(dropout_rate))
        model.add(BatchNormalization())
        model.add(Dense(1, activation='sigmoid'))
        model.compile(optimizer=optimizer,
                      loss='binary_crossentropy',
                      metrics=[tf.keras.metrics.AUC(curve='PR')])
        return model

    # perform k-fold cross validation, scored based on average precision
    kfold = StratifiedKFold(n_splits=n_folds, shuffle=True, random_state=5)
    print(f'Initializing MLP with {optimizer} optimizer, {activation} activation func, and {dropout_rate} dropout_rat
    es = EarlyStopping(monitor='auprc', mode='min', verbose=1, patience=10)
    nn = KerasClassifier(create_mlp, epochs=20, batch_size=300)
    score = cross_val_score(nn,
                            x_train,
                            y_train,
                            scoring='average_precision',
                            cv=kfold,
                            fit_params={'callbacks':[es],
                                        'class_weight':class_weights},
                            n_jobs=-1,
                            verbose=0).mean()

    res.append([optimizer, activation, dropout_rate, score])
    print(f'Finished iteration {i+1} of {n_iters} with precision score of {score}\n')
  return np.array(res)

# run experiments to determine model architecture
print('Running CV on model architecture')
model_arch_res = run_cv(20, 5)
```

```
## run k-fold cross validation to tune batch size and number of epochs to train

def run_cv2(n_iters, n_folds):
  """
  Custom function to tune number of epochs and batch size.
  Scores each fold based on average precision, to account for class imbalance.
  ====
  INPUTS
  - n_iters (integer): number of combinations to test
  - n_folds (integer): number of folds to run cross-validation
  OUTPUT
  - res: list of average precision scores
  """
  res = []
  for i in range(n_iters):
    # select random combination of optimizer, activation fn, and dropout rate
    n_epochs = np.random.choice([5, 10, 20, 30, 40, 50])
    bsize = np.random.choice([100, 150, 200, 250, 300, 250, 400, 450, 500])

    # create MLP model
    def create_mlp():
        model = Sequential()
        model.add(Dense(128, input_dim=x_train.shape[1], activation='relu'))
        model.add(Dropout(0.4))
        model.add(BatchNormalization())
        model.add(Dense(64, activation='relu'))
        model.add(Dropout(0.4))
        model.add(BatchNormalization())
        model.add(Dense(1, activation='sigmoid'))
        model.compile(optimizer='adam',
                      loss='binary_crossentropy',
                      metrics=[tf.keras.metrics.AUC(curve='PR')])
        return model

    # perform k-fold cross validation, scored based on average precision
    kfold = StratifiedKFold(n_splits=n_folds, shuffle=True, random_state=5)
    print(f'Initializing MLP with and training using {n_epochs} epochs and batch size of {bsize}')
    es = EarlyStopping(monitor='auprc', mode='min', verbose=1, patience=10)
    nn = KerasClassifier(create_mlp, epochs=20, batch_size=300)
    score = cross_val_score(nn,
                            x_train,
                            y_train,
                            scoring='average_precision',
                            cv=kfold,
                            fit_params={'callbacks':[es],
                                        'class_weight':class_weights},
                            n_jobs=-1,
                            verbose=0).mean()

    res.append([n_epochs, bsize, score])
    print(f'Finished iteration {i+1} of {n_iters} with precision score of {score}\n')
  return np.array(res)

print('\nRunning CV on batch size and number of epochs')
n_batchs_epochs_res = run_cv2(40, 5)


# get best hyperparameters using highest average precision

model_arch_cv_res = model_arch_res[model_arch_res[:, 3].argsort()[::-1]]
batches_epochs_results = n_batchs_epochs_res[n_batchs_epochs_res[:, 2].argsort()[::-1]]

best_params = {
    'optimizer':model_arch_cv_res[0,0],
    'activation':model_arch_cv_res[0,1],
    'dropout_rate':model_arch_cv_res[0,2],
    'n_epochs':int(batches_epochs_results[0,0]),
    'batch_size':int(batches_epochs_results[0,1])
}
best_params
```

```
# refit MLP using best hyperparameters
model = tf.keras.Sequential([
                     Dense(128, activation=best_params['activation']),
                     Dropout(best_params['dropout_rate']),
                     BatchNormalization(),
                     Dense(64, activation=best_params['activation']),
                     Dropout(best_params['dropout_rate']),
                     BatchNormalization(),
                     Dense(1, activation='sigmoid')])
model.compile(optimizer= best_params['optimizer'],
              loss='binary_crossentropy',
              metrics=[tf.keras.metrics.Recall(thresholds=0.5),
                       tf.keras.metrics.Precision(thresholds=0.5),
                       tf.keras.metrics.SpecificityAtSensitivity(sensitivity=0.80),
                       tf.keras.metrics.AUC(),
                       tf.keras.metrics.AUC(curve='PR'),
                       "accuracy"]
             )

es = EarlyStopping(monitor='auprc', mode='min', verbose=1, patience=10)
%time model.fit(x_train, y_train, epochs=best_params['n_epochs'], batch_size=best_params['batch_size'], callbacks=[es

# predict class probabilities
y_pred = model.predict_proba(x_test)
```

Discrimination Performance

```
## evaluate discrimination performance

# report discrimination metrics at different probability cutoffs
# (positive label assigned if positive class probability > cutoff)

cutoffs = np.arange(0.1, 1, 0.1)
target_names = ['negative', 'positive']

for cutoff in cutoffs:
  print('Cutoff: ', round(cutoff, 2))
  print(classification_report(y_test, np.where(y_pred[:,1] > cutoff, 1, 0), target_names=target_names))


# get roc
y_pos = y_pred[:, 1]
fpr, tpr, thresholds = roc_curve(y_test, y_pos)

# get index of sensitivity closest to 90+
indices = np.where(tpr >= 0.9)[0]
i = indices[tpr[indices].argmin()]

# calculate metrics
auroc = auc(fpr, tpr)
sens = tpr[i]*100
spec = (1 - fpr[i])*100
cutoff = thresholds[i]
y_pred_bin = (y_pred[:,1] >= cutoff).astype(int)
acc = accuracy_score(y_test, y_pred_bin)*100
avg_prec = average_precision_score(y_test, y_pos)*100
f1 = f1_score(y_test, y_pred_bin)*100

print("Recall of at least 90:  ", round(sens, 3))
print("Specificity:\t\t", round(spec,3))
print("AUROC:\t\t\t", round(auroc, 3))
print("Probabitiy cutoff:\t", round(cutoff, 3))
print('Accuracy:\t\t', round(acc, 3))
print('Average Precision:\t', round(avg_prec, 3))
print('F1-score:\t\t', round(f1, 3))
```

```
# plot ROC curve
plt.plot(fpr, tpr, label='Linear Classifier (AUROC = %0.2f)' % (auroc))
plt.xlim([-0.05, 1.05])
plt.ylim([-0.05, 1.05])
plt.axhline(y=sens/100, color='r', linestyle='--', linewidth=1)
plt.axvline(x=1-spec/100, color='r', linestyle='--', linewidth=1)
plt.xlabel('False positive rate')
plt.ylabel('True positive rate (Recall)')
plt.legend(loc="lower right")
plt.show()


# plot Precision-Recall curve
precision, recall, _ = precision_recall_curve(y_test, y_pos)
indeces = np.where(recall >= 0.9)[0]
i = indeces[recall[indeces].argmin()]
rec_at_cutoff = recall[i]*100
prec_at_cutoff = precision[i]*100

plt.plot(recall, precision, label='linear classifier (AP = %0.2f)' % (avg_prec))
plt.xlim([-0.05, 1.05])
plt.ylim([-0.05, 1.05])
plt.axhline(y=prec_at_cutoff/100, color='r', linestyle='--', linewidth=1)
plt.axvline(x=rec_at_cutoff/100, color='r', linestyle='--', linewidth=1)
plt.xlabel('Recall')
plt.ylabel('Precision')
plt.legend(loc="lower right")
plt.show()
```

XGBoost (Gradient Boosted Decision Tree)

```
import xgboost.sklearn as xgb


# define params for cross validation
param_grid = {
        'learning_rate': [0.01, 0.1],
        'max_depth': [3, 5],
        'subsample': [0.5, 0.8],
        'alpha': [0, 1],
        'objective': ['binary:logistic'],
        'eval_metric': ['aucpr'],
        'early_stopping_rounds': [10],
        'n_estimators': [300, 500],
        'random_state': [5],
        'tree_method': ['hist']
        }

# define model and CV
clf = xgb.XGBClassifier(scale_pos_weight=scale_pos_weight)
cv = StratifiedKFold(5, shuffle=True, random_state=5)
xgboost = GridSearchCV(clf, param_grid, cv = cv, error_score='raise', refit = True, verbose=True, n_jobs = -1, scorir

# tune and refit on best parameters
%time xgboost.fit(x_train, y_train)


# print best hyperparameters
best_params = xgboost.best_params_
best_params

# predict class probabilities
y_pred = xgboost.predict_proba(x_test)
```

Discrimination Performance

```
## evaluate discrimination performance

# report discrimination metrics at different probability cutoffs
# (positive label assigned if positive class probability > cutoff)

cutoffs = np.arange(0.1, 1, 0.1)
target_names = ['negative', 'positive']

for cutoff in cutoffs:
  print('Cutoff: ', round(cutoff, 2))
  print(classification_report(y_test, np.where(y_pred[:,1] > cutoff, 1, 0), target_names=target_names))


# get roc
y_pos = y_pred[:, 1]
fpr, tpr, thresholds = roc_curve(y_test, y_pos)

# get index of sensitivity closest to 90+
indices = np.where(tpr >= 0.9)[0]
i = indices[tpr[indices].argmin()]

# calculate metrics
auroc = auc(fpr, tpr)
sens = tpr[i]*100
spec = (1 - fpr[i])*100
cutoff = thresholds[i]
y_pred_bin = (y_pred[:,1] >= cutoff).astype(int)
acc = accuracy_score(y_test, y_pred_bin)*100
avg_prec = average_precision_score(y_test, y_pos)*100
f1 = f1_score(y_test, y_pred_bin)*100

print("Recall of at least 90:  ", round(sens, 3))
print("Specificity:\t\t", round(spec,3))
print("AUROC:\t\t\t", round(auroc, 3))
print("Probabitiy cutoff:\t", round(cutoff, 3))
print('Accuracy:\t\t', round(acc, 3))
print('Average Precision:\t', round(avg_prec, 3))
print('F1-score:\t\t', round(f1, 3))



# plot ROC curve
plt.plot(fpr, tpr, label='Linear Classifier (AUROC = %0.2f)' % (auroc))
plt.xlim([-0.05, 1.05])
plt.ylim([-0.05, 1.05])
plt.axhline(y=sens/100, color='r', linestyle='--', linewidth=1)
plt.axvline(x=1-spec/100, color='r', linestyle='--', linewidth=1)
plt.xlabel('False positive rate')
plt.ylabel('True positive rate (Recall)')
plt.legend(loc="lower right")
plt.show()


# plot Precision-Recall curve
precision, recall, _ = precision_recall_curve(y_test, y_pos)
indeces = np.where(recall >= 0.9)[0]
i = indeces[recall[indeces].argmin()]
rec_at_cutoff = recall[i]*100
prec_at_cutoff = precision[i]*100

plt.plot(recall, precision, label='linear classifier (AP = %0.2f)' % (avg_prec))
plt.xlim([-0.05, 1.05])
plt.ylim([-0.05, 1.05])
plt.axhline(y=prec_at_cutoff/100, color='r', linestyle='--', linewidth=1)
plt.axvline(x=rec_at_cutoff/100, color='r', linestyle='--', linewidth=1)
plt.xlabel('Recall')
plt.ylabel('Precision')
plt.legend(loc="lower right")
plt.show()
```

Model Evaluation

In this section we evaluated our highest performing model XGBoost. We analyze the following

- discrimination performance on resampled data
- predictor importance using SHapley Additive exPlanations
- calibration performance

XGBoost using resampled data

```
## create resampled data
from imblearn.combine import SMOTEENN                          # combine under-sampling and over sampling
from imblearn.under_sampling import EditedNearestNeighbours   # under-sample majority class
from imblearn.over_sampling import SMOTE                       # over-sample minority class


# define SMOTE-ENN
resample = SMOTEENN(enn=EditedNearestNeighbours(sampling_strategy='majority'),smote=SMOTE(sampling_strategy='minority
x_res, y_res = resample.fit_resample(x_train, y_train)

print('Original dataset shape %s' % Counter(y_train))
print('Resampled dataset shape %s' % Counter(y_res))


# define params for cross validation
param_grid = {
        'learning_rate': [0.01, 0.1],
        'max_depth': [3, 5],
        'subsample': [0.5, 0.8],
        'alpha': [0, 1],
        'objective': ['binary:logistic'],
        'eval_metric': ['aucpr'],
        'early_stopping_rounds': [10],
        'n_estimators': [300, 500],
        'random_state': [5],
        'tree_method': ['hist']
        }

# define model and CV
clf = xgb.XGBClassifier(scale_pos_weight=scale_pos_weight)
cv = StratifiedKFold(5, shuffle=True, random_state=5)
model = GridSearchCV(clf, param_grid, cv = cv, error_score='raise', refit = True, verbose=True, n_jobs = -1, scoring

# tune and refit on best parameters
%time model.fit(x_res, y_res)


# print best hyperparameters
best_params = model.best_params_
best_params

# predict class probabilities
y_pred = model.predict_proba(x_test)
```

Discrimination Performance

```
## evaluate discrimination performance

# report discrimination metrics at different probability cutoffs
# (pos label assigned if pos class probability > cutoff)

cutoffs = np.arange(0.1, 1, 0.1)
target_names = ['negative', 'positive']

for cutoff in cutoffs:
  print('Cutoff: ', round(cutoff, 2))
  print(classification_report(y_test, np.where(y_pred[:,1] > cutoff, 1, 0), target_names=target_names))


# get roc
y_pos = y_pred[:, 1]
fpr, tpr, thresholds = roc_curve(y_test, y_pos)

# get index of sensitivity closest to 90+
indices = np.where(tpr >= 0.9)[0]
i = indices[tpr[indices].argmin()]

# calculate metrics
auroc = auc(fpr, tpr)
sens = tpr[i]*100
spec = (1 - fpr[i])*100
cutoff = thresholds[i]
y_pred_bin = (y_pred[:,1] >= cutoff).astype(int)
acc = accuracy_score(y_test, y_pred_bin)*100
avg_prec = average_precision_score(y_test, y_pos)*100
f1 = f1_score(y_test, y_pred_bin)*100

print("Recall of at least 90:  ", round(sens, 3))
print("Specificity:\t\t", round(spec,3))
print("AUROC:\t\t\t", round(auroc, 3))
print("Probabitiy cutoff:\t", round(cutoff, 3))
print('Accuracy:\t\t', round(acc, 3))
print('Average Precision:\t', round(avg_prec, 3))
print('F1-score:\t\t', round(f1, 3))


# plot ROC curve
plt.plot(fpr, tpr, label='Linear Classifier (AUROC = %0.2f)' % (auroc))
plt.xlim([-0.05, 1.05])
plt.ylim([-0.05, 1.05])
plt.axhline(y=sens/100, color='r', linestyle='--', linewidth=1)
plt.axvline(x=1-spec/100, color='r', linestyle='--', linewidth=1)
plt.xlabel('False positive rate')
plt.ylabel('True positive rate (Recall)')
plt.legend(loc="lower right")
plt.show()


# plot Precision-Recall curve
precision, recall, _ = precision_recall_curve(y_test, y_pos)
indeces = np.where(recall >= 0.9)[0]
i = indeces[recall[indeces].argmin()]
rec_at_cutoff = recall[i]*100
prec_at_cutoff = precision[i]*100

plt.plot(recall, precision, label='linear classifier (AP = %0.2f)' % (avg_prec))
plt.xlim([-0.05, 1.05])
plt.ylim([-0.05, 1.05])
plt.axhline(y=prec_at_cutoff/100, color='r', linestyle='--', linewidth=1)
plt.axvline(x=rec_at_cutoff/100, color='r', linestyle='--', linewidth=1)
plt.xlabel('Recall')
plt.ylabel('Precision')
plt.legend(loc="lower right")
plt.show()
```

```
import shap # shapley additive explanations

# SHAP feature importance
explainer = shap.TreeExplainer(model.best_estimator_)
shap_values = explainer.shap_values(x_test)
shap.summary_plot(shap_values, x_test, show=False)
```
